# Determinants of emergency presentation in patients with colorectal cancer: a systematic review and meta-analysis

**DOI:** 10.1038/s41598-022-08447-y

**Published:** 2022-03-14

**Authors:** Allan M. Golder, Donald C. McMillan, Paul G. Horgan, Campbell S. D. Roxburgh

**Affiliations:** Academic Unit of Surgery, Department of Surgery, University of Glasgow, Level 2, New Lister Building, Glasgow Royal Infirmary, Glasgow, G31 2ER UK

**Keywords:** Cancer epidemiology, Cancer, Gastrointestinal cancer, Colorectal cancer

## Abstract

Colorectal cancer remains a significant cause of morbidity and mortality, even despite curative treatment. A significant proportion of patients present emergently and have poorer outcomes compared to elective presentations, independent of TNM stage. In this systematic review and meta-analysis, differences between elective/emergency presentations of colorectal cancer were examined to determine which factors were associated with emergency presentation. A literature search was carried out from 1990 to 2018 comparing elective and emergency presentations of colon and/or rectal cancer. All reported clinicopathological variables were extracted from identified studies. Variables were analysed through either systematic review or, if appropriate, meta-analysis. This study identified multiple differences between elective and emergency presentations of colorectal cancer. On meta-analysis, emergency presentations were associated with more advanced tumour stage, both overall (OR 2.05) and T/N/M/ subclassification (OR 2.56/1.59/1.75), more: lymphovascular invasion (OR 1.76), vascular invasion (OR 1.92), perineural invasion (OR 1.89), and ASA (OR 1.83). Emergencies were more likely to be of ethnic minority (OR 1.58). There are multiple tumour/host factors that differ between elective and emergency presentations of colorectal cancer. Further work is required to determine which of these factors are independently associated with emergency presentation and subsequently which factors have the most significant effect on outcomes.

## Introduction

Colorectal cancer is the third most commonly diagnosed malignancy worldwide with approximately 1.1 million cases of colon cancer and 700,000 cases of rectal cancer being diagnosed each year^1^. Combined, these account for around 860,000 deaths per year. The National Bowel Cancer Audit 2017^2^ reported that 75% of those patients diagnosed with colorectal cancer in England and Wales undergo curative treatment though, despite this, a significant number of these patients succumb from their disease. Large bowel obstruction is currently the 4th most common indication for emergency laparotomy in the United Kingdom accounting for 14.4% of emergency laparotomies performed^3^ with colorectal malignancy likely to be the main underlying pathology.

The route to diagnosis and surgical treatment of cancer has multiple sub-classifications^4^ but can be broadly classified as elective or emergency. While the majority of colorectal cancer presents electively, a significant minority—10–30% presents as an emergency^5–8^. Despite many countries introducing a colorectal cancer screening program, the rate of emergency presentation remains high. Within the United Kingdom, the proportion of colorectal cancer presenting emergently remains at 20%^9^.

There is an association between emergency presentations of colorectal cancer and significantly worse short- and long-term outcomes. While factors including more advanced disease stage and higher American Society of Anaesthesiology (ASA) Grade at presentation may contribute to this, recent research suggests that emergency presentation remains an independent poor prognostic indicator following curative colorectal resection^10,11^.

It is likely that the worse outcomes observed in emergency compared to elective presentations of colorectal cancer are due to disparities in tumour and host factors between modes of presentation rather than being due to emergency presentation per se. To improve long-term outcomes within this high-risk group of emergency patients it is essential to firstly determine how elective and emergency patients differ both in terms of tumour factors and host factors and subsequently to determine which of these factors have the most significant effect on long-term outcomes. For common clinicopathological factors the association between these factors and mode of presentation have been previously studied. For other, more novel clinicopathological factors, the association with mode of presentation may yet to be studied. To the best of our knowledge, to date, the existing literature comparing mode of presentation and clinicopathological factors has yet to be comprehensively summarised.

The present systematic review and meta-analysis aims to comprehensively review thirty years of literature analysing the association between clinicopathological factors and mode of presentation of colorectal cancer to identify those factors that differ between elective and emergency presentations of colorectal cancer.

## Methods

This systematic review and meta-analysis of published literature was carried out according to a pre-defined protocol. The primary outcome was to compare the differences between tumour factors and host factors and mode of presentation of colorectal cancer.

Studies published between January 1990 and August 2018 were identified through an electronic search of the US National Library of Medicine (MEDLINE) and the Cochrane Database of Systematic Reviews. Selected other studies were identified through a manual bibliography search. The following search strategy was used: (colon OR rectum OR rectal OR colorectal) AND (cancer OR carcinoma OR adenocarcinoma OR neoplasm OR malign OR tumour) AND (emergency OR acute OR urgent OR non-elective) AND (surgery OR surgical OR operation OR resection OR procedure).

**Figure 1:**
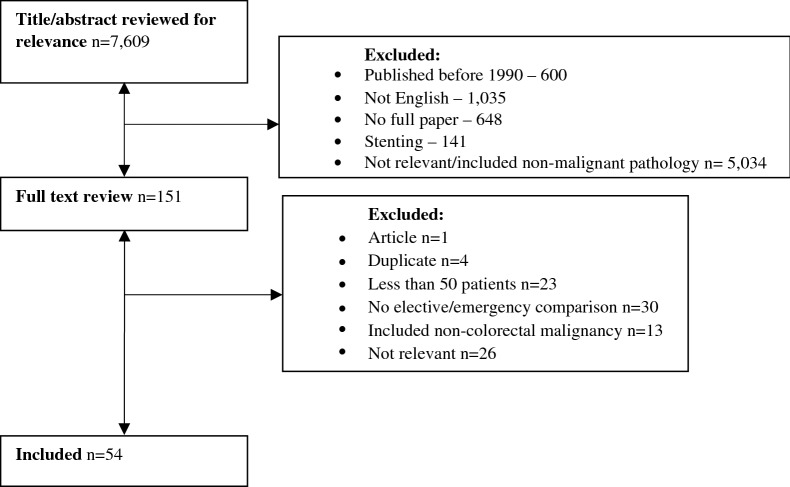
PRISMA statement.

On completion of the online search, the title and abstract of each identified study was examined for relevance with full text being obtained for all potentially relevant studies. This was undertaken by an individual researcher with discussion with a senior author if required. Studies were included regardless of design, with both trials and observational studies being eligible for inclusion. Studies that were not in English, studies where the full text was not available, studies that included patients undergoing colorectal resection for pathology other than cancer or patients undergoing colonic stenting were excluded. The present study involved a wide literature search to capture as much of the pre-existing literature as possible however small studies (deemed those with less than 50 patients within the emergency group) were excluded to reduce the risk of bias. In those instances where multiple studies were available using the same patient population only the most recent study was included. If populations varied the most inclusive study was used. Those studies that did not provide comparison between elective and emergency patients were excluded from this review. This is shown in our PRISMA flow diagram (Fig. 1).

Provided there were 3 or more studies for a particular factor, a meta-analysis of tumour/host factors was performed. Papers included either reported the numbers of emergency and elective patients and the number of patients with the factor of interest analysed or reported percentages in a way that allowed these numbers to be calculated. The Cochrane Handbook for Systematic Reviews^12^ has been used to guide the reporting of results within the present study.

### Statistical analysis

All statistical analyses were performed using Review Manager (RevMan) Version 5.3, The Cochrane Collaboration. For all comparisons an unadjusted odds ratio was used. Where possible, total sample sizes and events were taken from the raw data presented in each study. If events were reported as a percentage of total sample size, the event size was calculated from this percentage. 95% confidence intervals were used throughout and a p value of < 0.05 was considered to be significant. Forest plots were used for graphical display of results. The degrees of heterogeneity were defined as non-significant between 0 and 30%, moderate between 30 and 50%, substantial between 50 and 75% and considerable between 75 and 100%

## Results

### Literature search

Studies were selected as demonstrated in the PRISMA diagram (Fig. 1). The initial search strategy identified 7,609 studies whose titles and abstracts were reviewed. Studies were excluded that were published prior to 1990 (n = 600), not in English (n = 1,035), primarily compared colonic stenting (n = 141), did not have an available full paper (n = 648) or were either not relevant to this topic or included pathologies other than colorectal cancer (n = 5,034). This led to the review of 151 full papers. Of these a further 97 were excluded as they included less than 50 patients (n = 23), did not provide a comparison between elective and emergency patients (n = 30), included pathologies other than colorectal cancer (n = 13), were articles (n = 1), duplicate studies (n = 4) or were not relevant (n = 26). The remaining 54 studies were included in this review.

### Tumour factors

#### Tumour location

20 studies examined the association between tumour location and mode of presentation in 97,788 patients (Supplementary Table S1). Within this review, tumours of the right colon, hepatic flexure and transverse colon were considered right sided. Tumours of the splenic flexure, left colon and sigmoid colon have been considered left sided. Rectosigmoid and rectal tumours have been considered rectal.

**Figure 2:**
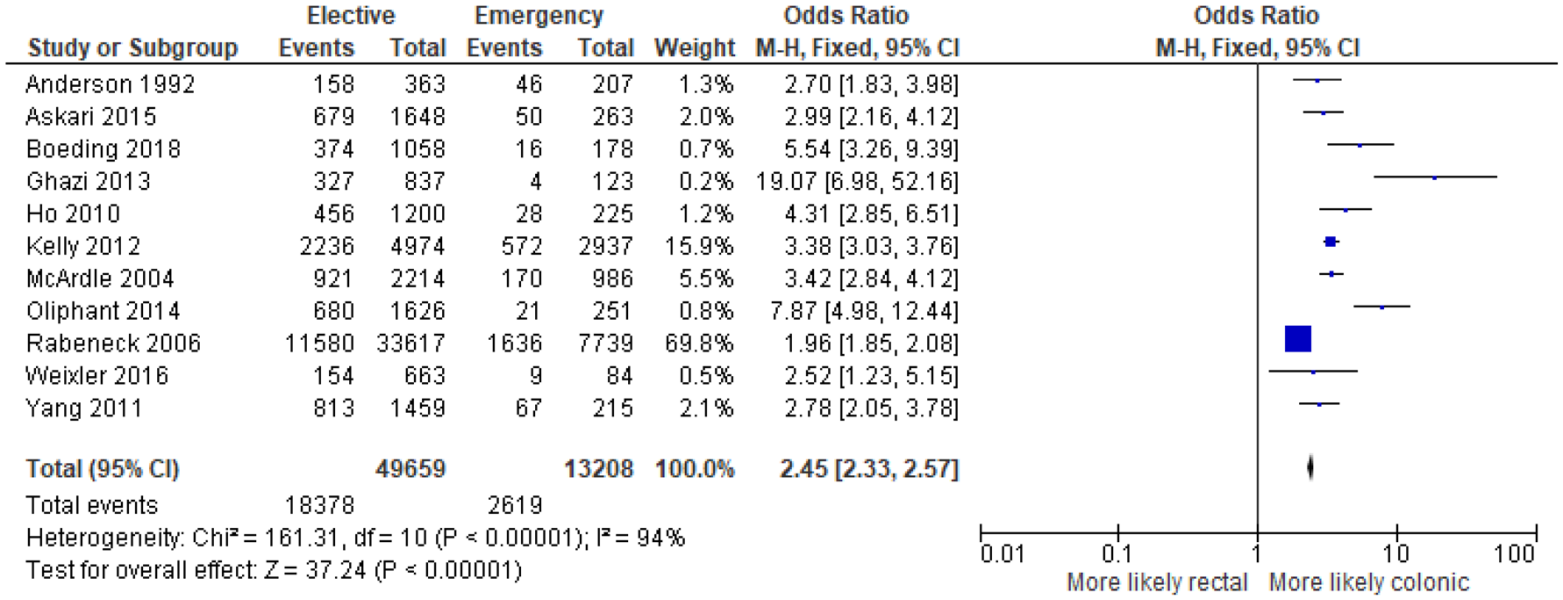
Association between tumour location (rectal vs colonic) and emergency presentation—Forest Plot.

11 studies^7,13–23^ examined the association between colonic/rectal location and mode of presentation in 62,867 patients. On meta-analysis including all of these studies (Fig. 2) there was an association between emergency presentation and colonic location (OR 2.45, 95% CI 2.33–2.57, P < 0.001, I^2^ = 94%).

**Figure 3:**
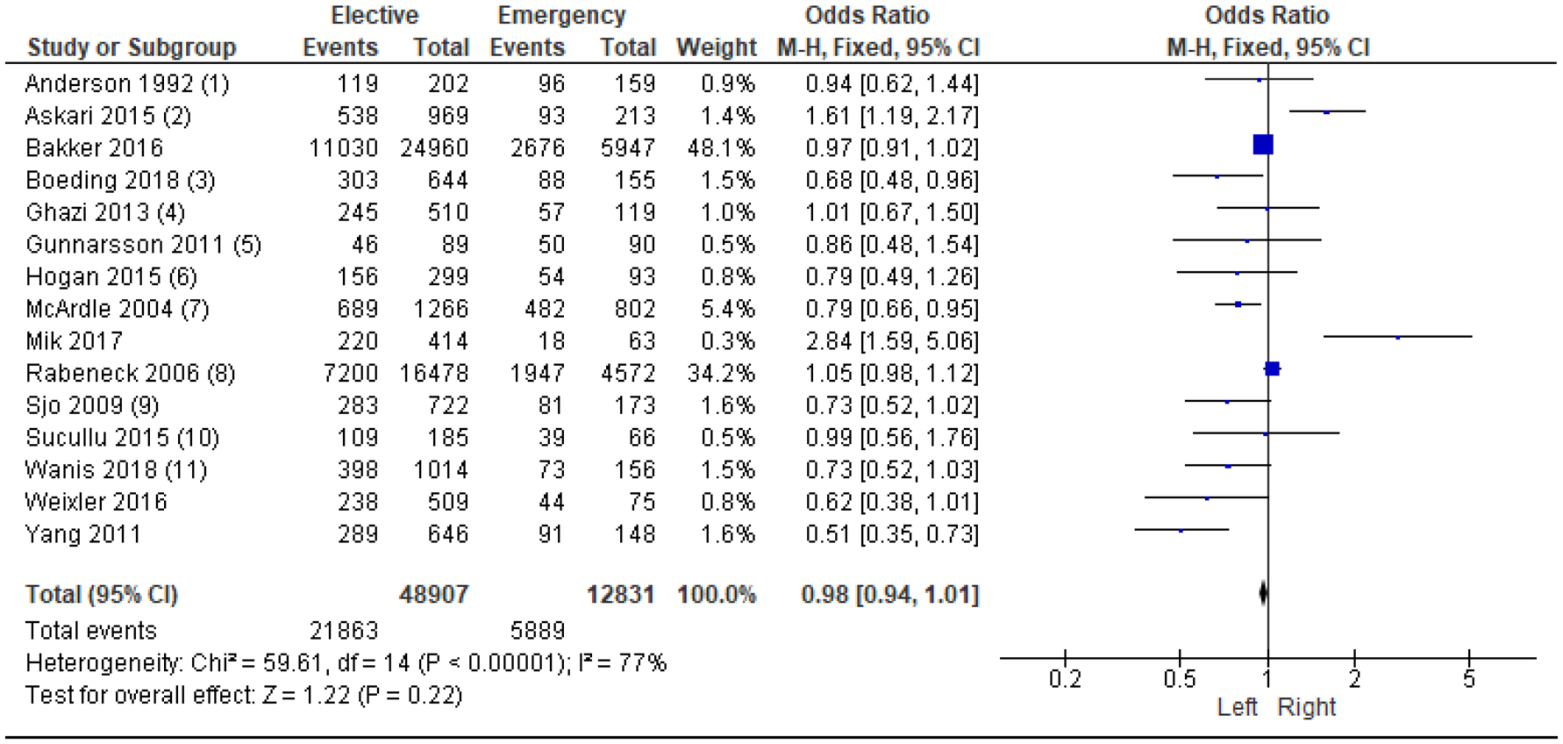
Association between colonic tumour location (right sided vs left sided) and emergency presentation—Forest Plot.

19 studies^7,13–19,21–31^ examined the association between colonic location (left/right) and mode of presentation in 95,911 patients. On meta-analysis including 15 studies of 61,738 patients (Fig. 3) no significant association was reported between emergency presentation and colonic location (OR 0.98, 95% CI 0.94–1.01, P = 0.22, I^2^ = 77%).

#### Tumour size

1 study^15^ examined the association between tumour size and mode of presentation in 1,672 patients (Supplementary Table S2) and reported an association between emergency presentation and larger tumour diameter (p = 0.011).

#### Tumour staging

##### Overall staging

**Figure 4:**
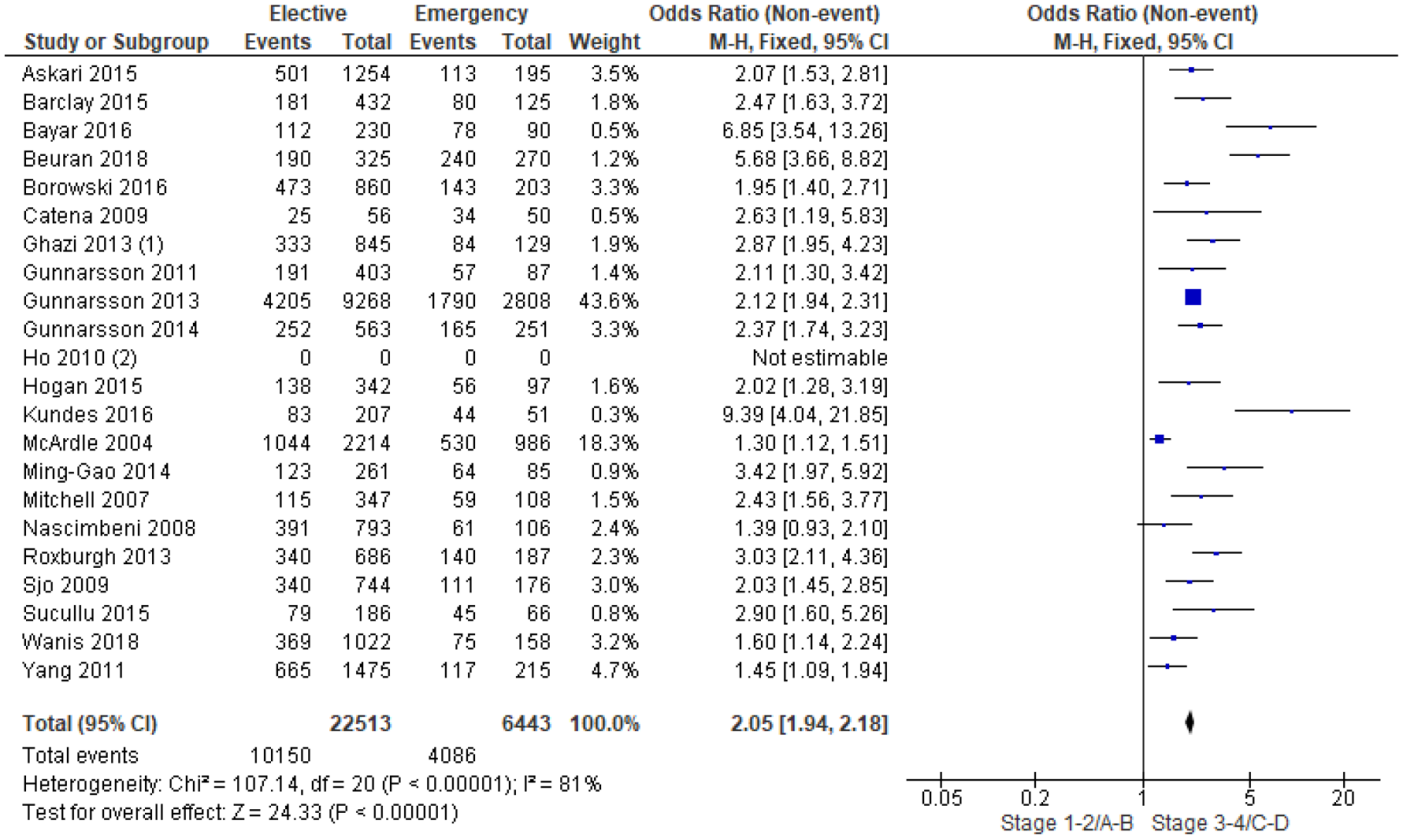
Association between overall tumour staging and emergency presentation—Forest plot.

22 studies^13,15,16,18,19,23–25,28,30–42^ examined the association between overall tumour stage (TNM/Dukes Staging (Table 1)) and mode of presentation in 30,382 patients (Supplementary Table S3). On meta-analysis including 21 studies of 28,956 patients (Fig. 4) there was an association between emergency presentation and more advanced (TNM 3–4) overall tumour stage (OR 2.05, 95% CI 1.94–2.18, P < 0.001, I^2^ = 81%).

**Table 1:** TNM and dukes staging.

TNM stage	TNM classification	Dukes classification
Stage 0	Tis, N0, M0	
Stage1	T1-2, N0, M0	A
Stage IIA	T3, N0, M0	B
Stage IIB	T4, N0, M0	B
Stage IIIA	T1-2, N1, M0	C
Stage IIIB	T3-4, N1, M0	C
Stage IIIC	Any T, N2, M0	C
Stage IV	Any T, Any N, M1	D

##### Tumour stage (T stage)

**Figure 5:**
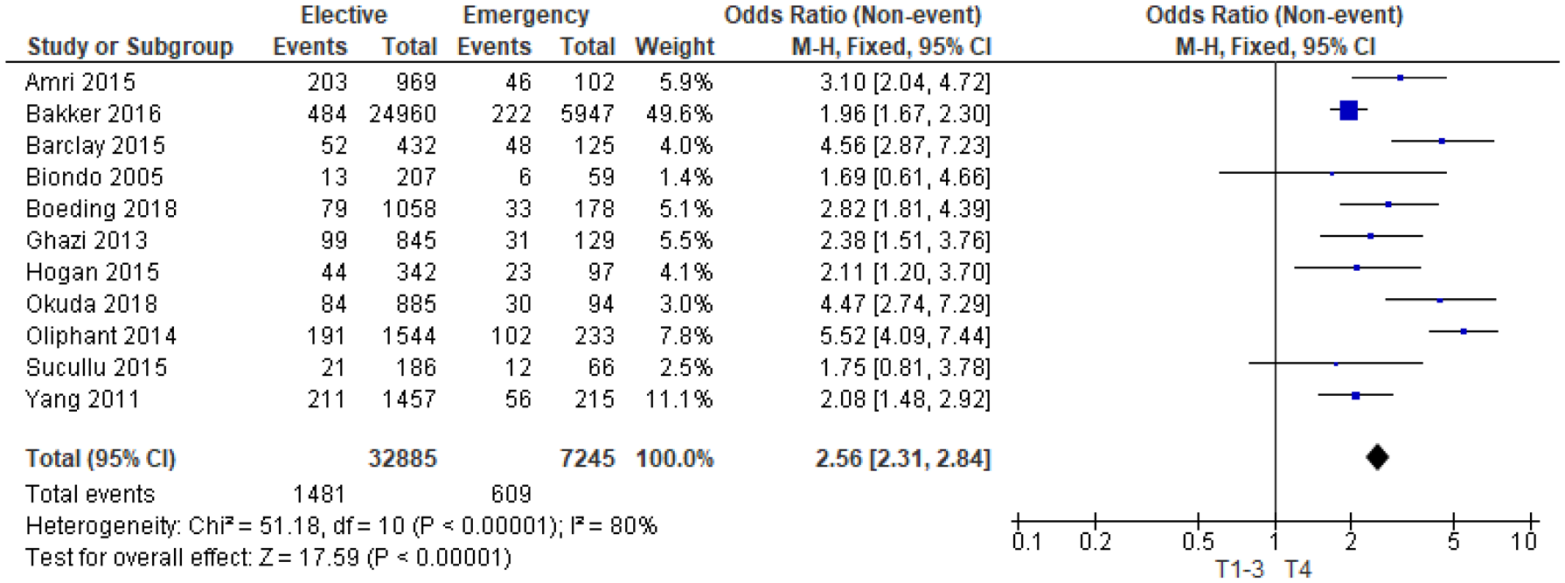
Association between T Stage and emergency presentation—Forest Plot.

11 studies^13,15,20,22,24,27–29,38,43,44^ examined the association between T Stage and mode of presentation in 40,130 patients (Supplementary Table S4). On meta-analysis including all of these studies (Fig. 5) there was a significant association between emergency presentation and T4 disease (OR 2.56, 95% CI 2.31–2.84, P < 0.001, I^2^ = 80%).

##### Nodal stage (N stage)

**Figure 6:**
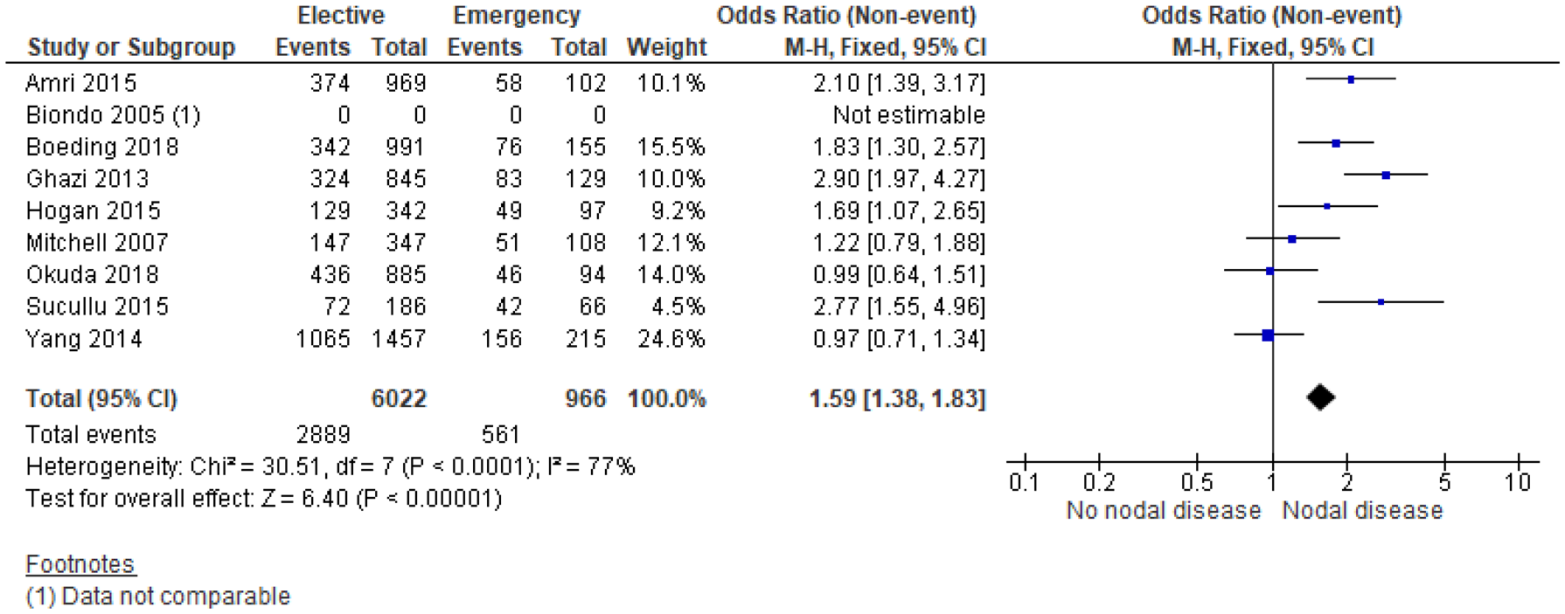
Association between N Stage and emergency presentation—Forest Plot.

9 studies^13,22,24,25,27,28,33,43,44^ examined the association between N Stage and mode of presentation in 7,254 patients (Supplementary Table S5). On meta-analysis including 8 studies of 6,988 patients (Fig. 6) there was an association between emergency presentation and node positive disease (OR 1.59, 95% CI 1.38–1.83, P < 0.001, I^2^ = 77%).

##### Metastatic disease (M stage)

**Figure 7:**
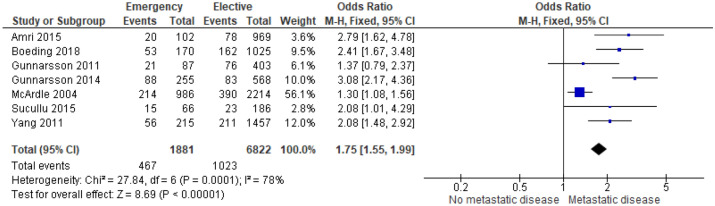
Association between M Stage and emergency presentation—Forest Plot.

7 studies^15,19,22,24,25,35,43^ examined the association between M Stage and mode of presentation in 8,703 patients (Supplementary Table S6). On meta-analysis including all of these studies (Fig. 7) there was an association between emergency presentation and metastatic disease (OR 1.75, 95% CI 1.55–1.99, P < 0.001, I^2=^78%).

#### Histological features

##### Tumour circumference

1 study^25^ examined the association between luminal tumour circumference and mode of presentation in 150 patients (Supplementary Table S7) and reported an association between emergency presentation and tumour circumference of greater than two thirds of the luminal circumference (p = 0.009).

##### Tumour type

4 studies^13,15,18,45^ examined the association between tumour type and mode of presentation in 84,791 patients (Supplementary Table S8). One study^45^ of 81,825 patients found an inverse association between emergency presentation and simple adenocarcinomas (83% vs 85%) and an association between emergency presentation and proportion of mucinous/signet type tumours (12% vs 11%) however it was unclear whether this was of statistical significance. Two studies^15,18^ of 1992 patients reported no significant association between emergency presentation and histological tumour type.

##### Lymphovascular invasion

**Figure 8:**
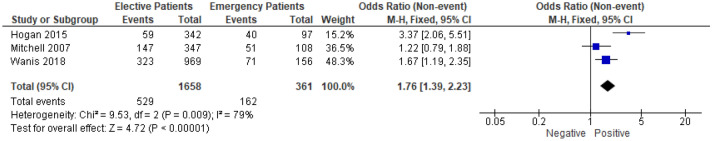
Association between presence of lymphovascular invasion and emergency presentation—Forest Plot.

3 studies^28,30,33^ examined the association between lymphovascular invasion and mode of presentation in 2,019 patients (Supplementary Table S9). On meta-analysis including all of these studies (Fig. 8) there was an association between emergency presentation and lymphovascular invasion (OR 1.76, 95% CI 1.39–2.23, P < 0.001, I^2^ = 79%).

##### Vascular invasion

**Figure 9:**
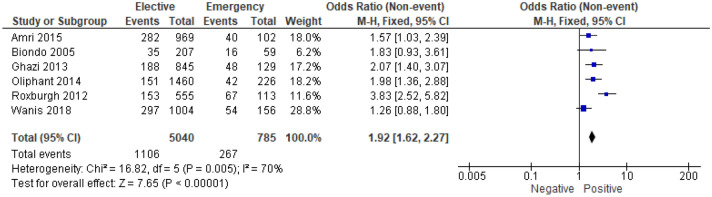
Association between presence of vascular invasion and emergency presentation—Forest Plot.

6 studies^13,20,27,30,36,43^ examined the association between vascular invasion and mode of presentation in 5,825 patients (Supplementary Table S10). On meta-analysis including all of these studies (Fig. 9) there was an association between emergency presentation and vascular invasion (OR 1.92, 95% CI 1.62–2.27, P < 0.001, I^2^ = 70%).

##### Tumour perforation

1 study^36^ examined the association between tumour perforation and the mode of presentation in 707 patients (Supplementary Table S11) and reported an association between emergency presentation and microscopic perforation (P = 0.010).

##### Perineural invasion

**Figure 10:**
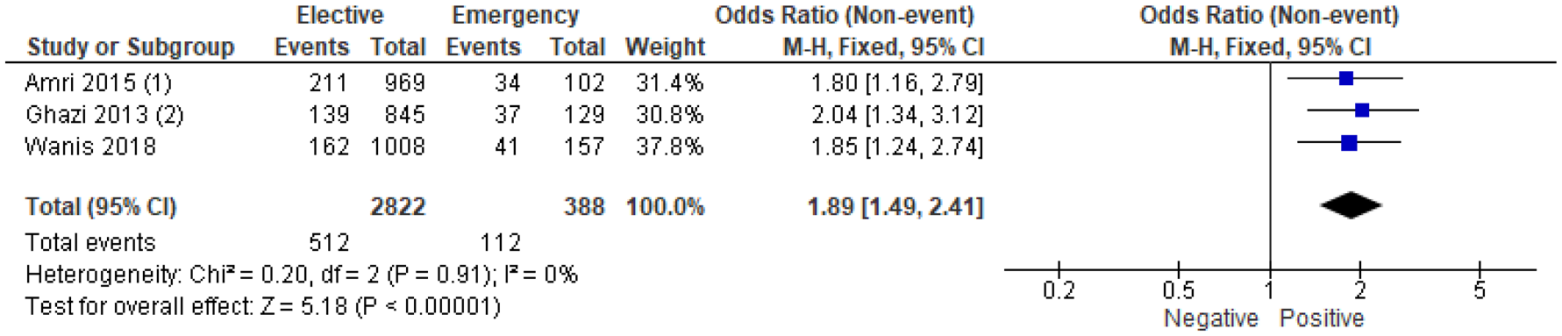
Association between presence of perineural invasion and emergency presentation—Forest Plot.

3 studies^13,30,43^ examined the association between perineural invasion and mode of presentation in 3210 patients (Supplementary Table S12). On meta-analysis including all of these studies (Fig. 10) there was an association between emergency presentation and perineural invasion (OR 1.89, 95% CI 1.49–2.41, P < 0.001, I^2^ = 0%).

##### Tumour desmoplasia, necrosis and budding

1 study^13^ examined the association between tumour desmoplasia (Supplementary Table S13), necrosis (Supplementary Table S14) and budding (Supplementary Table S15) and mode of presentation in 974 patients. Tumour desmoplasia was associated with emergency presentations (OR 2.11, P = 0.03). No significant association was reported between emergency presentation and either tumour necrosis or tumour budding (P = 0.33 and P = 0.28 respectively).

##### Tumour differentiation/grade

**Figure 11:**
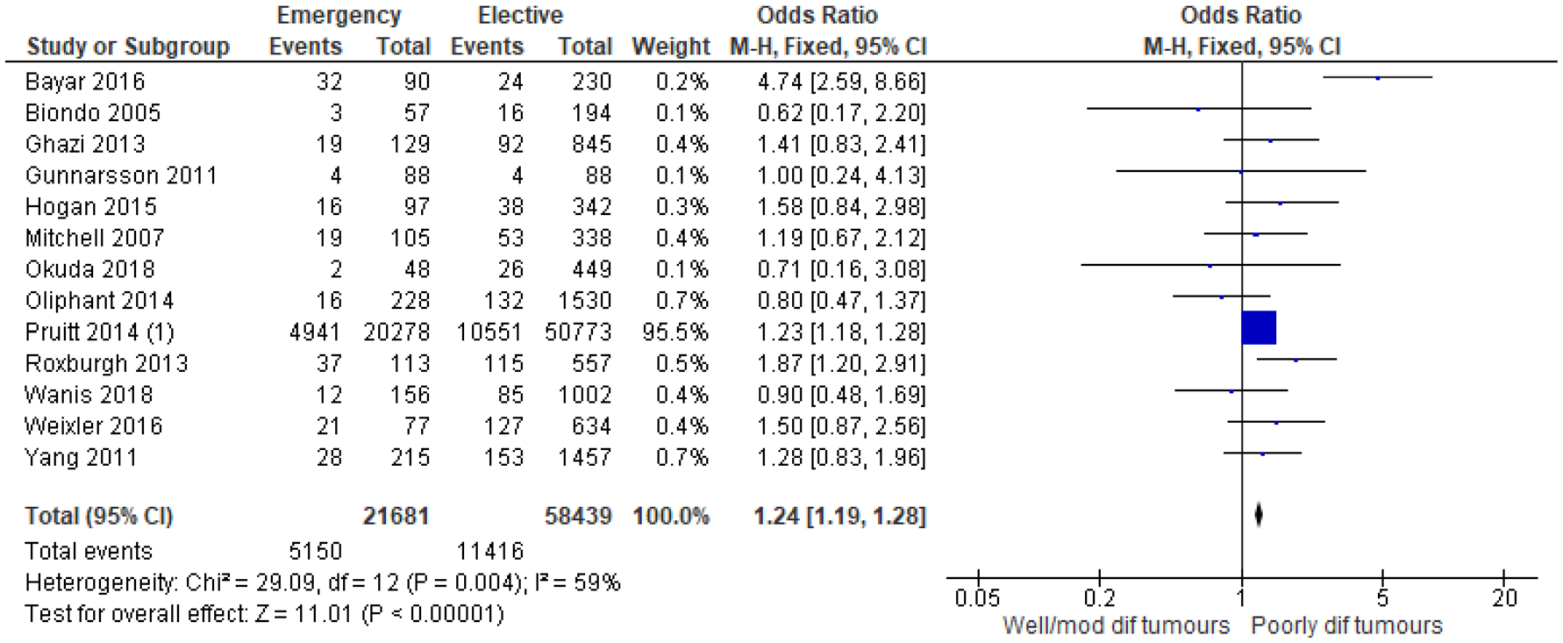
Association between tumour grade/differentiation and emergency presentation—Forest Plot.

13 studies^7,13,15,18,20,25,27,28,30,33,36,44,45^ examined the association between tumour differentiation/grade and mode of presentation in 80,626 patients (Supplementary Table S16). On meta-analysis including all of these studies (Fig. 11) there was an association between emergency presentation and high grade/poorly differentiated tumours (OR 1.24, 95% CI 1.19–1.28, P < 0.001, I^2^ = 59%).

### Host factors

#### Sex

**Figure 12:**
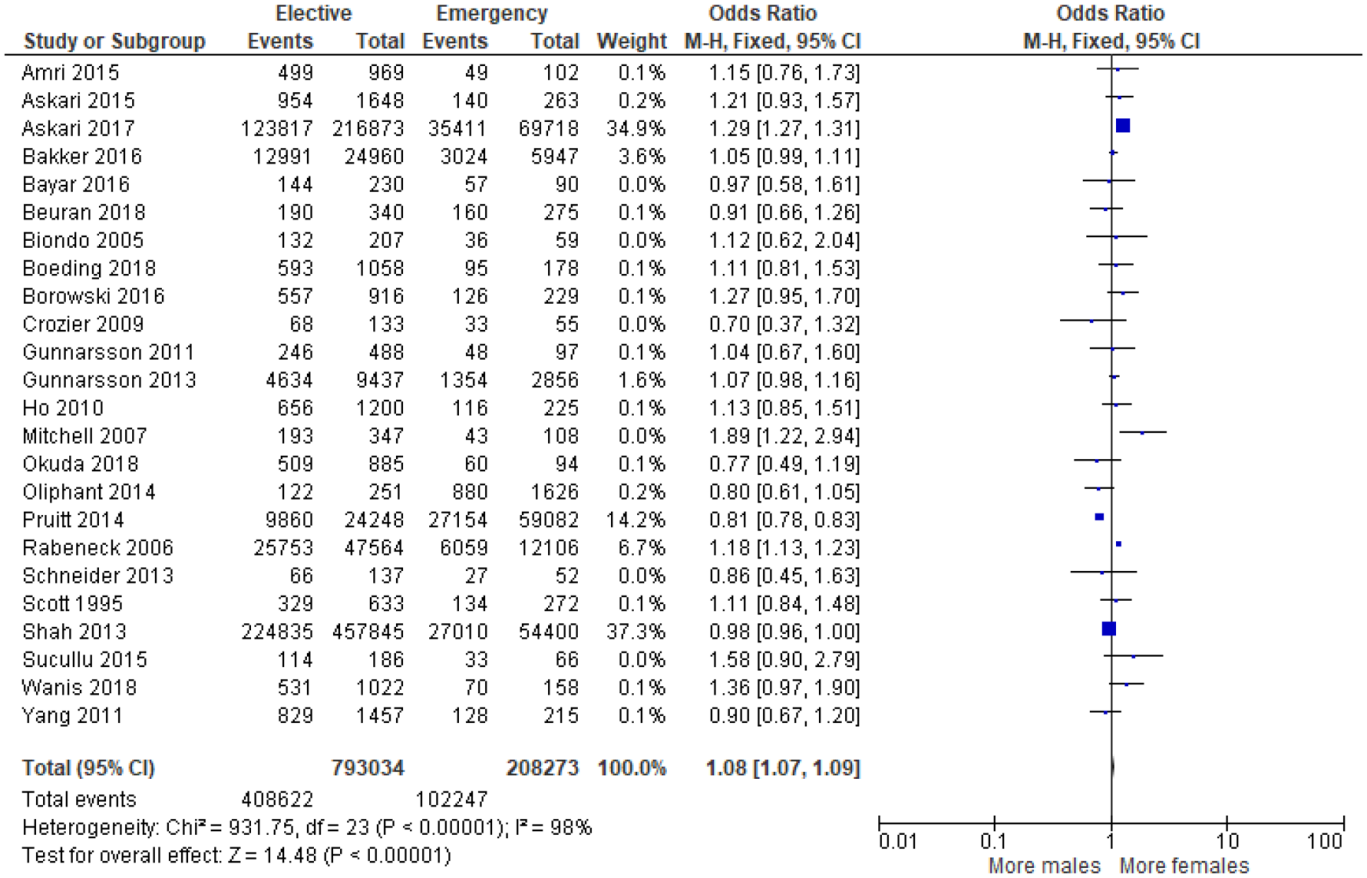
Association between sex and emergency presentation—Forest Plot.

24 studies^15,16,18,20,22–25,27,29,30,32,33,37,41,43–51^ examined the association between patient sex and mode of presentation in 1,001,307 (Supplementary Table S17). On meta-analysis that included all of these studies (Fig. 12) there was an association between emergency presentation and female sex (OR 1.08, 95% CI 1.07–1.09, P < 0.001, I^2^ = 98%).

#### Age

29 studies^5,14,15,17–20,24,25,27,29,30,32–37,39–41,43,44,46–48,51–53^ examined the association between age and mode of presentation in 909,131 patients (Supplementary Table S18). Due to heterogeneity of data it was not possible to perform a meta-analysis of this factor.

11 studies of 514,205 patients did not find a significant association between emergency presentation and age. This included a large study^48^ from the USA of 507,750 patients that compared the proportion of patients aged over 65 who presented either electively or as an emergency. 18 studies of 394,926 patients found an association between emergency presentation and older age. This included a study^51^ from the UK of 286,591 patients (P < 0.001). 10 studies^5,14,17,19,29,32,36,46,51,52^ subcategorised age into < 70/70 + (n = 1), < 75/75 + (n = 6) and < 80/80 + (n = 3) in 386,618 patients. 9 studies of 386,430 patients found an association between emergency presentation and older age.

### Ethnicity

**Figure 13:**
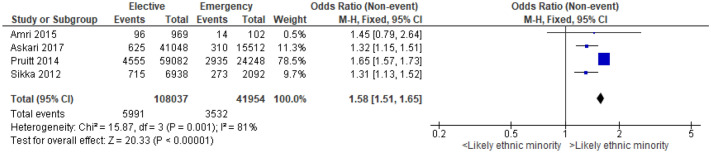
Association between ethnicity and emergency presentation—Forest Plot.

4 studies^5,43,45,51^ examined the association between ethnicity and mode of presentation in 149,991 patients (Supplementary Table S19). Three of these studies were from the USA and one was from the UK. Two studies compared white vs African-American individuals, one study classified patients as either White, Black or Asian and the final study classified patients as ethnic minority (yes/no) however did not provide further description of ethnic minority status. On meta-analysis including all of these studies (Fig. 13) there was an association between emergency presentation and ethnic minority status (OR 1.58, 95% CI 1.51–1.65, I^2^ = 81%).

### Body mass index

3 studies^33,43,54^ examined the association between Body Mass Index (BMI) and mode of presentation in 1,700 patients (Supplementary Table S20). Two studies^43,54^ of 1071 patients reported no significant association between emergency presentation and median BMI. One study^33^ of 455 patients reported an association between a BMI < 25 or > 40 and emergency presentation (P = 0.001).

### Distance to hospital

1 study^55^ examined the association between distance to hospital and mode of presentation in 380 patients (Supplementary Table S21)—no significant association was found.

#### Socioeconomic status

14 studies^14,16,32,33,36,37,45–47,51,55–58^ examined the association between socioeconomic status and mode of presentation in 433,364 (Supplementary Table S22). Due to heterogeneity of data it was not possible to perform a meta-analysis of this factor.

6 studies^14,32,37,45,51,56^ of 426,348 patients reported an association between emergency presentation and socio-economic deprivation. This included a study of 284,235 patients from the UK that classified patients into S.I.M.D. quintiles—emergency surgery was more likely in the most deprived quintile (Quintile 1 → Quintile 5 OR 1.64, 95% CI 1.50–1.80).

#### Comorbid status

##### ASA grade

**Figure 14:**
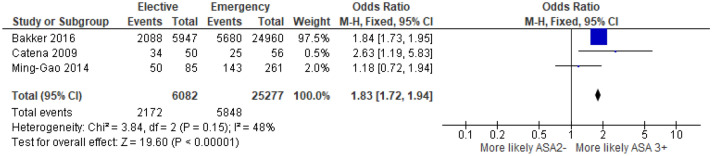
Association between ASA Grade and emergency presentation—Forest Plot.

3 studies^29,39,42^ examined the association between ASA grade and mode of presentation in 31,359 patients (Supplementary Table S23). On meta-analysis including all of these studies (Fig. 14) there was an association between emergency presentation and ASA ≥ 3 (OR 1.83, 95% CI 1.72–1.94, P < 0.001, I^2^ = 48%).

##### Other assessments of comorbidity

11 studies^5,15,16,18,29,35,43,48,49,59,60^ examined the association between co-morbid status and mode of presentation in 724,136 patients (Supplementary Table S24). Co-morbidities were compared using a variety of methods that included Charlson Score, Comorbidities (Yes/No) or the presence of specific co-morbidities including diabetes, cardiovascular or respiratory disease. Due to heterogeneity of data it was not possible to perform a meta-analysis of this factor.

2 studies of 538,939 patients^29,48^ reported an association between emergency presentation and less co-morbid status. This included a study^48^ of 508,032 patients that reported a Charlson Score ≥ 2 in 8.6% of emergency patients and 9.2% of elective patients (p ≤ 0.001). A further study^29^ of 30,907 patients reported a Charlson score of ≥ 2 in 24% of emergency patients and 26% of elective patients (level of statistical significance not provided).

7 studies^5,14–16,18,59,60^ of 183,286 patients reported an association between emergency presentation and more co-morbid status.

#### Pre-operative systemic inflammatory response

2 studies^39,61^ examined the association between pre-operative systemic inflammatory response and mode of presentation in 1246 patients (Supplementary Table S25). 1 study reported both the modified Glasgow Prognostic Score^62^ (mGPS) and Neutrophil–Lymphocyte ratio^63^ (NLR) and 1 study reported preoperative C-reactive protein (CRP). Both studies reported an association between emergency presentation and the preoperative systemic inflammatory response.

### Seasonal variability

1 study^25^ examined the association between seasonal variability and mode of presentation (Supplementary Table S26) and reported an association between emergency presentation and presentation during the summer months (June–August) in comparison to the winter months (December-February)—36% vs 23% P = 0.05.

### Other factors

1 study^64^ examined the association between haemoglobin and weight loss and mode of presentation in 372 patients (Supplementary Table S27). Low haemoglobin levels and weight loss were both associated with emergency presentation (both P ≤ 0.001).

1 study^39^ examined the association between CEA, TNF A, IL1 and IL6 and mode of presentation in 106 patients (Supplementary Table S28) and reported a significantly higher CEA, IL1 and IL6 in the emergency cohort. No significant difference was reported in TNF A levels.

## Discussion

The present systematic review and meta-analysis confirms multiple differences in tumour, host and other factors between elective and emergency presentations of colorectal cancer. It may therefore be a combination of these factors that are associated with the poorer short- and long-term outcomes reported in emergency presentations of colorectal cancer^10,11^ rather than emergency presentation per se.

**Figure 15:**
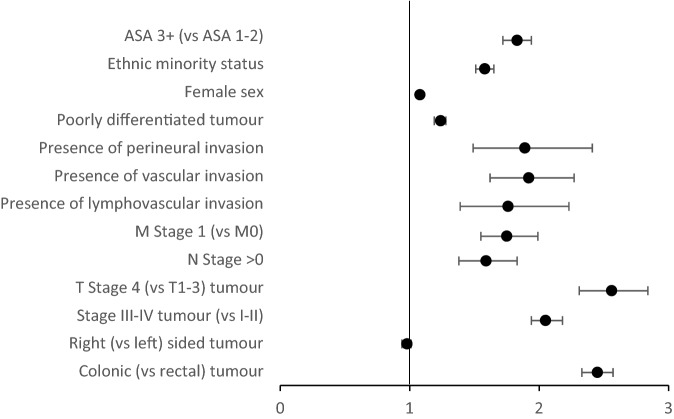
Summary of meta-analysis findings—association between clinicopathological characteristics and emergency presentation (odds rations and 95% confidence intervals).

In particular, tumour location (colon vs rectum), tumour stage, lymphovascular/perineural invasion, tumour differentiation, ethnicity and ASA grade differed significantly on meta-analysis between the elective and emergency cohorts as summarised in Fig. 15. Although not analysed in the meta-analysis due to study heterogeneity/< 3 studies other factors that differed between elective and emergency presentations include age, socioeconomic status and the preoperative systemic inflammatory response. Many of these factors have been reported to be associated with oncological outcomes in colorectal cancer^38,65–68^ and it therefore cannot be assumed that the negative effect of emergency presentation is solely due to more advanced disease. More recently, factors including body composition^69^ and perioperative blood transfusion^70^ have been reported to be associated with poorer long-term outcomes following curative resection for colorectal cancer and would be of interest for inclusion in future studies comparing elective and emergency presentations. The present review found that, on meta-analysis, ethnic minority status was associated with emergency presentation. However, given that the included studies were either from the USA or UK, non-Caucasian was essentially considered the ethnic minority group. No studies compared the effect of ethnic minority status in a country where Caucasian was the minority group and this would be an interesting area of future research.

Emergency presentations of colorectal cancer remain associated with poorer long-term outcomes than elective presentations, even after adjustment for TNM stage. Indeed, within TNM Stage II colorectal cancer, emergency presentation is considered to be a high-risk factor requiring consideration for adjuvant chemotherapy^71–73^. Further research would allow for both adjusted analysis of factors associated with emergency presentation and the subsequent effect of these on long-term outcomes both within the overall patient population and within stage-specific disease.

Over the last two decades, colorectal cancer screening programs have become widespread throughout the developed world. While participation in screening programs has resulted in a significant reduction in the proportion of patients presenting emergently^74^ many patients continue to present with acute symptoms requiring emergency investigation and treatment. The present review included literature from both a screening and pre-screening era. It has been shown that factors including age, sex, socioeconomic status and tumour stage and site^75^ differ between unscreened patents and those patients who have either participated in or been diagnosed through screening. No studies have been identified to date comparing emergency presentations between those patients who did/did not participate in screening and this would be of interest in future work.

The present study has several limitations. Due to the nature of this study, a significant degree of heterogeneity was present both in terms of inclusion criteria and reported outcomes within individual studies. Therefore, it was not possible to compare adjusted data hence the use of unadjusted data within the present review. Factors within the present study including age and BMI have not been included within meta-analysis due to data heterogeneity and the continuous nature of these variables. Consideration was given to conducting meta-regression however in keeping with guidance^12^ this could not be carried out due to the small number of studies suitable for such analysis. While the present review identified a large number of studies comparing elective and emergency presentations of colorectal cancer, very few studies subclassify emergency presentations into their presenting diagnoses, predominantly obstruction, perforation and bleeding. It therefore remains uncertain how factors and outcomes vary between different emergency presentations. One would hypothesise that patients presenting with perforation may have significantly different characteristics and outcomes than those presenting with an otherwise uncomplicated large bowel obstruction. The optimal management of patients presenting as an emergency with large bowel obstruction remains uncertain. While the majority of patients undergo emergency colonic resection, some clinicians opt for primary colonic stenting in the emergency setting with subsequent elective resectional surgery. This in an important question which remains unanswered however lies outside the scope of the present review^76–78^. It is commonplace within Systematic Reviews and Meta-Analyses to present risk of bias and quality of included studies using a variety of measures^12^. However the nature of the present review does not analyse the effect of an intervention on outcomes and therefore such measures are not applicable to the present review. Furthermore, with reference to specific factors, the small number of studies precluded meaningful analysis of the overall quality of studies and risk of bias.

In summary, the present study has identified multiple factors that differ between elective and emergency presentations of colorectal cancer as reported within the past 30 years of literature. This literature review paves the way to determining which tumour and host factors are independently significant with mode of presentation and which have the most significant effects on short- and long-term outcomes therefore explaining the poorer outcomes reported within emergency presentations. Defining these factors would help to determine those patients that have the worst short-term and long-term outcomes and therefore identify strategies within the perioperative and adjuvant settings to improve outcomes for these high-risk patients.

## Supplementary Information

Supplementary Tables.
