# Supplementary material for: Determinants of emergency presentation in patients with colorectal cancer: a systematic review and meta-analysis
Source: Sci Rep. 2022 Mar 14;12:4366. doi: 10.1038/s41598-022-08447-y (PMC8921241; doi:10.1038/s41598-022-08447-y)
Supplement: Supplementary file 1 — Supplementary Tables. [file 41598_2022_8447_MOESM1_ESM.docx]

**Supplementary Tables**

| **Study** | **n – El**  **(n - Em)** | **Country**  **Years** | **Location**  **Stage** | **Cohort** | **Comparison** | **Outcome** | **P Value** |
| --- | --- | --- | --- | --- | --- | --- | --- |
| Sucullu^(24)^  2015 | 186  (66) | Turkey  2004-2013 | Colon  TNM I-IV | Curative resections | Colon location | No sig dif | 0.818 |
| Ghazi^(13)^  2013 | 837  (123) | Sweden  2004-2006 | Colorectal  TNM I-IV | All resections | Colon/rectum | Association between colonic and Em | <0.001 |
|  |  |  |  |  | Colon location | Association between left sided and Em | - |
| Rabeneck^(14)^  2006 | 33617  (7739) | Canada  1996-2001 | Colorectal  TNM I-IV | All cases | Colon/rectum | Association between colonic and Em | <0.001 |
|  |  |  |  |  | Colon location | 44% of elective left side vs 43% Em | - |
| Yang^(15)^  2011 | 1459  (215) | China  1998-2005 | Colorectal  TNM I-IV | All surgery | Colon/rectum | Association between colonic and Em | <0.001 |
|  |  |  |  |  | Colon location | Association between > left sided and Em | <0.001 |
| Askari^(16)^  2015 | 1648  (263) | UK  2004-2014 | Colorectal  TNM I-IV | All surgery | Colon/rectum | Association between colonic and Em | <0.001 |
| Anderson^(17)^  1992 | 363  (207) | UK  1974-1979 | Colorectal  TNM I-IV | All cases | Colon/rectum | Association between colonic and Em | - |
|  |  |  |  |  | Colon location | Association between left sided and Em | - |
| Gunnarsson^(25)^  2011 | 89  (90) | Sweden  1996-2005 | Colon  TNM I-IV | All resections | Colon location | Association between left sided and Em | 0.04 |
| Mik^(26)^  2017 | 414  (63) | Poland  2009-2012 | Colon  TNM I-IV | All resections | Colon location | Association between left sided and Em | 0.006 |
| Biondo^(27)^  2005 | 207  (59) | Spain  1996-1998 | Colon  TNM I-III | Curative resections | Colon location | No sig dif | 1 |
| Bayar^(18)^  2016 | 230  (90) | Turkey  2009-2013 | Colorectal  TNM I-IV | All surgery | Colon/rectum | Association between colonic and Em | - |
|  |  |  |  |  | Colon location | Association between left sided and Em | - |
| McArdle^(19)^  2004 | 2214  (986) | UK  1991-1994 | Colorectal  TNM I-IV | All resections | Colon/rectum | Association between colonic and Em | - |
|  |  |  |  |  | Colon location | Association between left sided and Em | - |
| Oliphant^(20)^  2014 | 1626  (251) | UK  2001-2004 | Colorectal  TNM I-II | Curative resections | Colon/rectum | Association between colonic and Em | <0.001 |
| Hogan^(28)^  2015 | 342  (97) | Ireland  2000-2010 | Colon  TNM I-IV | Curative resections | Colon location | Rectosigmoid Em<El  SF and desc Em>El | 0.004 |
| Kelly ^(21)^  2012 | 4974  (2937) | Ireland  2002-2008 | Colorectal  TNM I-IV | All resections | Colon/rectum | Association between colonic and Em | X |
|  |  |  |  |  | Colon location | Right Em>El | X |
| Bakker^(29)^  2016 | 24960  (5947) | Netherlands  2009-2013 | Colon TNM I-IV | All resections | Colon location | Association between left sided and Em | X |
| Boeding^(22)^  2018 | 1058  (178) | Netherlands  2004-2010 | Colorectal  TNM I-IV | All resections | Colon/rectum | Association between colonic and Em | <0.1 |
|  |  |  |  |  | Colon location | Association between left sided and Em | - |
| Wanis^(30)^  2018 | 1022  (158) | Canada  2006-2015 | Colon  TNM I-III | Curative resections | Colon location | Association between left sided and Em | X |
| Sjo^(31)^  2009 | 740  (170) | Norway  1993-2007 | Colon  TNM I-IV | All surgery | Colon location | No sig dif | 0.12 |
| Ho^(23)^  2010 | 1193  (223) | Australia  1984-2004 | Colorectal  TNM I-III | Curative resections | Colon/rectum | Association between colonic and Em | <0.001 |
|  |  |  |  |  | Colon location | Association between left sided and Em | Significant |
| Weixler^(7)^  2016 | 663  (84) | Switzerland  1989-2013 | Colorectal  TNM I-IV | All resections | Colon/rectum | Association between colonic and Em | 0.019 |
|  |  |  |  |  | Colon location | Association between left sided and Em | - |

*Supplementary Table 1 – Association between tumour location (colonic vs rectal and colonic location) and mode of presentation*

| **Study** | **n – El**  **(n - Em)** | **Country**  **Years** | **Location**  **Stage** | **Cohort** | **Comparison** | **Outcome** | **P Value** |
| --- | --- | --- | --- | --- | --- | --- | --- |
| Yang^(15)^  2011 | 1457  (215) | China  1998-2005 | Colorectal  TNM I-IV | All surgery | Tumour size <5cm less or >5cm | Association between >5cm tumours and Em | 0.011 |

*Supplementary Table 2 – Association between tumour size and mode of presentation*

| **Study** | **n – El**  **(n - Em)** | **Country**  **Years** | **Location**  **Stage** | **Cohort** | **Comparison** | **Outcome** | **P Value** |
| --- | --- | --- | --- | --- | --- | --- | --- |
| Sucullu^(24)^  2015 | 186  (66) | Turkey  2004-2013 | Colon  TNM I-IV | All resections | TNM | Association between > TNM Stage and Em | <0.001 |
| Ghazi^(13)^  2013 | 845 (129) | Sweden  2004-2006 | Colorectal  TNM I-IV | All resections | TNM | Association between > TNM Stage and Em | <0.0001 |
| Yang^(15)^  2011 | 1475 (215) | China  1998-2005 | Colorectal  TNM I-IV | All surgery | TNM | Association between > TNM Stage and Em | 0.016 |
| Askari^(16)^  2015 | 1254 (195) | UK  2004-2014 | Colorectal  TNM I-IV | All surgery | TNM | Association between > TNM Stage and Em | <0.001 |
| Gunnarsson^(32)^  2013 | 9286 (2808) | Sweden  1997-2006 | Colon  TNM I-IV | All cases | TNM | Association between > TNM Stage and Em | <0.001 |
| Gunnarsson^(25)^  2011 | 403  (87) | Sweden  1997-2006 | Colon  TNM I-IV | All resections | TNM | Association between > TNM Stage and Em | <0.001 |
| Mitchell^(33)^  2007 | 347 (108) | Canada  2002-2004 | Colorectal  TNM I-IV | All resections | TNM | Association between > TNM Stage and Em | <0.001 |
| Nascimbeni^(34)^  2008 | 793  (106) | Italy  1975-1984 | Colorectal  TNM I-IV | All surgery | Dukes | Association between > Dukes Stage and Em | 0.01 |
| Gunnarsson^(35)^  2014 | 563 (251) | Sweden  2006-2008 | Colon  TNM I-IV | All surgery | TNM | Association between > TNM Stage and Em | <0.001 |
| Roxburgh^(36)^  2013 | 686  (187) | UK  2001-2010 | Colorectal  TNM I-IV | All cases | TNM | Association between > TNM Stage and Em | <0.001 |
| Borowski^(37)^  2016 | 860  (203) | UK  2009-2014 | Colorectal  TNM I-IV | All cases | TNM | Association between > TNM Stage and Em | <0.001 |
| Barclay^(38)^  2015 | 432  (125) | Australia  2005-2010 | Colorectal  TNM I-IV | All resections | TNM | Association between > TNM Stage and Em | <0.001 |
| Catena^(39)^  2009 | 106  (50) | Italy  1999-2001 | Colon and high rectal  TNM I-IV | All resections | Dukes | Association between > Dukes Stage and Em | <0.05 |
| Bayar^(18)^  2016 | 320  (90) | Turkey  2009-2013 | Colorectal  TNM I-IV | All surgery | TNM | Association between > TNM Stage and Em | <0.001 |
| McArdle^(19)^  2004 | 3200  (986) | UK  1991-1994 | Colorectal  TNM I-IV | All resections | Dukes | Association between > Dukes Stage and Em | <0.001 |
| Hogan^(28)^  2015 | 56  (50) | Ireland  2000-2010 | Colon  TNM I-IV | Curative resections | TNM | Association between > TNM Stage and Em | <0.01 |
| Kundes^(40)^  2016 | 207  (51) | Turkey  2012-2014 | Colorectal  TNM I-III | Curative resections | TNM | Association between > TNM Stage and Em | <0.0001 |
| Wanis^(30)^  2018 | 1022  (158) | Canada  2006-2015 | Colon  TNM I-III | Curative resections | TNM | Association between > TNM Stage and Em | - |
| Beuran^(41)^  2018 | 325 (270) | Romania  2011-2016 | Left colon  TNM I-IV | All resections | TNM | Association between > TNM Stage and Em | - |
| Sjo^(31)^  2009 | 744  (176) | Norway  1993-2007 | Colon  TNM I-IV | All surgery | TNM | Association between > TNM Stage and Em | <0.01 |
| Ho^(23)^  2010 | 1193 (233) | Australia  1984-2004 | Colorectal  TNM I-III | Curative resections | TNM | Association between > TNM Stage and Em | <0.01 |
| Ming-Gao^(42)^  2014 | 261  (85) | China  2000-2010 | Colorectal  TNM I-IV | All surgery | Dukes | Association between > Dukes Stage and Em | <0.01 |

*Supplementary Table 3 – Association between overall tumour staging (TNM/Dukes) and mode of presentation*

| **Study** | **n – El**  **(n - Em)** | **Country**  **Years** | **Location**  **Stage** | **Cohort** | **Comparison** | **Outcome** | **P Value** |
| --- | --- | --- | --- | --- | --- | --- | --- |
| Sucullu^(24)^  2015 | 186  (66) | Turkey  2004-2013 | Colon  TNM I-IV | Curative resections | T Stage 1/2/3/4 | Association between > T Stage and Em | <0.001 |
| Ghazi^(13)^  2013 | 845  (129) | Sweden  2004-2006 | Colorectal  TNM I-IV | All resections | T2 | No sig dif | 0.72 |
|  |  |  |  |  | T3 | More T3 in Em vs El | 0.03 |
|  |  |  |  |  | T4 | More T4 in Em vs El | 0.002 |
| Yang^(15)^  2011 | 1457  (215) | China  1998-2005 | Colorectal  TNM I-IV | All surgery | T Stage 1/2/3/4 | Association between >T Stage and Em | <0.0001 |
| Barclay^(38)^  2015 | 432  (125) | Australia  2005-2010 | Colorectal  TNM I-IV | All resections | T Stage 1/2/3/4 | Association between > T Stage and Em | <0.0001 |
| Amri^(43)^  2015 | 969  (102) | USA  2004-2011 | Colon  TNM I-IV | All resections | T Stage 1-3/4 | Association between > T Stage and Em | <0.001 |
| Biondo^(27)^  2005 | 207  (59) | Spain  1996-1998 | Colon  TNM I-III | Curative resections | T Stage 1/2/3/4 | Association between > T Stage and Em | 0.014 |
| Okuda^(44)^  2018 | 885  (94) | Japan  2007-2011 | Colorectal  TNM II-III | Curative resections | T Stage in TNM II | No sig difference | 0.652 |
|  |  |  |  |  | T Stage in TNM III | Association between > T Stage and Em | <0.001 |
| Oliphant^(20)^  2014 | 1544  (233) | UK  2001-2004 | Colorectal  TNM I-II | Curative resections | T Stage  1/2/3/4 | Association between > T Stage and Em | <0.001 |
| Hogan^(28)^  2015 | 342  (97) | Ireland  2000-2010 | Colon  TNM I-IV | Curative  Resections | T1 | Sig more el than em | 0.017 |
|  |  |  |  |  | T2 | Sig more el than em | 0.008 |
|  |  |  |  |  | T3 | No sig dif | 0.495 |
|  |  |  |  |  | T4 | Sig more em than el | 0.009 |
| Bakker^(29)^  2016 | 24960  (5947) | Netherlands  2009-2013 | Colon  TNM I-IV | All resections | T Stage  1-2/3/4 | Association between > T4 disease and Em | - |
| Boeding^(22)^  2018 | 1058  (178) | Netherlands  2004-2010 | Colorectal  TNM I-IV | All resections | T Stage 1/2/3/4 | Sig fewer T1/2 in Em than El | <0.01 |

*Supplementary Table 4 – Association between T stage and mode of presentation*

| **Study** | **n – El**  **(n - Em)** | **Country**  **Years** | **Location**  **Stage** | **Cohort** | **Comparison** | **Outcome** | **P Value** |
| --- | --- | --- | --- | --- | --- | --- | --- |
| Sucullu^(24)^  2015 | 186  (66) | Turkey  2004-2013 | Colon  TNM I-IV | Curative resections | N 0/N1-2 | Association between > N Stage and Em | 0.002 |
| Ghazi^(13)^  2013 | 845  (129) | Sweden  2004-2006 | Colorectal  TNM I-IV | All resections | N 0/1/2 | Association between > N Stage and Em | <0.001 |
| Yang^(15)^  2011 | 1457  (215) | China  1998-2005 | Colorectal  TNM I-IV | All surgery | N 0/1-2 | No sig dif | 0.868 |
|  |  |  |  |  | N 1/2 | No sig dif | 0.567 |
| Mitchell^(33)^  2007 | 347  (108) | Canada  2002-2004 | Colorectal  TNM I-IV | All resections | N 0/1-2 | No sig dif | 0.38 |
| Amri^(43)^  2015 | 969  (102) | USA  2004-2011 | Colon  TNM I-IV | All resections | N 0/1-2 | Association between > N Stage and Em | <0.001 |
| Biondo^(27)^  2006 | 207  (59) | Spain  1996-1998 | Colon  TNM I-III | Curative resections | N0/1-2 | No sig dif | 0.222 |
| Okuda^(44)^  2018 | 885  (94) | Japan  2007-2011 | Colorectal  TNM II-III | All resections | N1/2 in TNM III | No sig dif | 0.355 |
| Hogan^(28)^  2015 | 342  (97) | Ireland  2000-2010 | Colon  TNM I-IV | Curative resections | N0 el vs em | N0 – more El than Em | 0.016 |
|  |  |  |  |  | N1 el vs em | N1 – no sig dif | 0.527 |
|  |  |  |  |  | N2 el vs em | N2 – more Em than El | <0.01 |
| Boeding^(22)^  2018 | 991  (155) | Netherlands  2004-2010 | Colorectal  TNM I-IV | All resections | N0 el vs em | Assoc between <Em and N0 | <0.01 |
|  |  |  |  |  | N1 el vs em | Assoc between >N1 and Em | 0.02 |
|  |  |  |  |  | N2 el vs em | No sig dif | 0.048 |

*Supplementary Table 5 – Association between N stage and mode of presentation*

| **Study** | **n – El**  **(n - Em)** | **Country**  **Years** | **Location**  **Stage** | **Cohort** | **Comparison** | **Outcome** | **P Value** |
| --- | --- | --- | --- | --- | --- | --- | --- |
| Sucullu^(24)^  2015 | 186  (66) | Turkey  2004-2013 | Colon  TNM I-IV | Curative resections | Metastatic disease  Yes/No | Association between > metastatic disease and Em | 0.037 |
| Yang^(15)^  2011 | 1457  (215) | China  1998-2005 | Colorectal  TNM I-IV | All surgery | Liver mets  Yes/no | Association between > metastatic disease and Em | 0.001 |
| Gunnarsson^(25)^  2011 | 403  (87) | Sweden  1996-2005 | Colon  TNM I-IV | All resections | Metastatic disease  Yes/No | Association between > metastatic disease and Em | - |
| Gunnarsson^(35)^  2014 | 568  (255) | Sweden  2006-2008 | Colon  TNM I-IV | All surgery | Metastatic disease  Yes/No | Association between > metastatic disease and Em | - |
| Amri ^(43)^  2015 | 969  (102) | USA  2004-2011 | Colon  TNM I-IV | All resections | Metastatic disease  Yes/No | Association between > metastatic disease and Em | <0.001 |
| McArdle^(19)^  2004 | 2214  (986) | UK  1991-1994 | Colorectal  TNM I-IV | All resections | Metastatic disease  Yes/no | Association between > metastatic disease and Em | - |
| Boeding^(22)^  2018 | 1025  (170) | Netherlands  2004-2010 | Colorectal  TNM I-IV | All resections | Metastatic disease  Yes/No | Association between > metastatic disease and Em | <0.01 |

*Supplementary Table 6 – Association between presence of metastatic disease and mode of presentation*

| **Study** | **n – El**  **(n - Em)** | **Country**  **Years** | **Location**  **Stage** | **Cohort** | **Comparison** | **Outcome** | **P Value** |
| --- | --- | --- | --- | --- | --- | --- | --- |
| Gunnarsson^(25)^  2011 | 67  (83) | Sweden  1996-2005 | Colon  TNM I-IV | All resections | +/- 2/3 of luminal circumference | Association between circumference >2/3 and Em | 0.009 |

*Supplementary Table 7 – Association between luminal tumour circumference and mode of presentation*

| **Study** | **n – El**  **(n - Em)** | **Country**  **Years** | **Location**  **Stage** | **Cohort** | **Comparison** | **Outcome** | **P Value** |
| --- | --- | --- | --- | --- | --- | --- | --- |
| Ghazi^(13)^  2012 | 845  (129) | Sweden  2004-2006 | Colorectal  TNM I-IV | All resections | Mucin type | No sig dif | >0.05 |
|  |  |  |  |  | Signet ring type | Association between > Signet ring and Em | 0.001 |
| Yang^(15)^  2011 | 1457  (215) | China  1998-2005 | Colorectal  TNM I-IV | All surgery | Adeno vs mucinous vs signet ring tumours | No sig dif | 0.123 |
| Pruitt^(45)^  2014 | 58158  (23667) | USA  1992-2005 | Colorectal  TNM I-IV | All cases | Adenocarcinoma vs mucinuous/signet ring vs other | Association between non-simple adeno and Em | - |
| Bayar^(18)^  2016 | 230  (90) | Turkey  2009-2013 | Colorectal  TNM I-IV | All surgery | Adeno vs mucinous vs signet cell vs insitu vs malignant epithlelial tumour | No sig dif | >0.05 |

*Supplementary Table 8 – Association between tumour type and mode of presentation*

| **Study** | **n – El**  **(n - Em)** | **Country**  **Years** | **Location**  **Stage** | **Cohort** | **Comparison** | **Outcome** | **P Value** |
| --- | --- | --- | --- | --- | --- | --- | --- |
| Mitchell^(33)^  2007 | 347  (108) | Canada  2002-2004 | Colorectal  TNM I-IV | All resections | LVI  (Pos/neg) | Association between LVI and Em | 0.03 |
| Hogan^(28)^  2015 | 342  (97) | Ireland  2000-2010 | Colon  TNM I-IV | Curative resections | LVI  (Pos/neg) | Association between LVI and Em | <0.01 |
| Wanis^(30)^  2018 | 969  (156) | Canada  2006-2015 | Colon  TNM I-III | Curative resections | LVI  (Pos/neg) | Association between LVI and Em | - |

*Supplementary Table 9 – Association between presence of lymphovascular invasion (LVI) and mode of presentation*

| **Study** | **n – El**  **(n - Em)** | **Country**  **Years** | **Location**  **Stage** | **Cohort** | **Comparison** | **Outcome** | **P Value** |
| --- | --- | --- | --- | --- | --- | --- | --- |
| Ghazi^(13)^  2013 | 845  (129) | Sweden  2004-2006 | Colorectal  TNM I-IV | All resections | Vascular invasion | Association between > Vascular Invasion and Em | <0.001 |
| Roxburgh^(36)^  2013 | 555  (113) | UK  2001-2010 | Colorectal  TNM I-IV | cases | EMVI | Association between > Vascular Invasion and Em | <0.001 |
| Amri^(43)^  2015 | 969  (102) | USA  2004-2011 | Colon  TNM I-IV | All resections | EMVI | Association between > Vascular Invasion and Em | 0.021 |
| Biondo^(27)^  2005 | 207  (59) | Spain  1996-1998 | Colorectal  TNM I-III | Curative resections | Vascular invasion | No sig dif | 0.092 |
| Oliphant^(20)^  2014 | 1460  (226) | UK  2001-2004 | Colorectal  TNM I-II | Curative resections | EMVI | Association between > Vascular Invasion and Em | 0.001 |
| Wanis^(30)^  2018 | 1004  (156) | Canada  2006-2015 | Colon  TNM I-III | Curative resections | Venous invasion | Association between > Vascular Invasion and Em | x |

*Supplementary Table 10 -Association between presence of vascular invasion and mode of presentation*

| **Study** | **n – El**  **(n - Em)** | **Country**  **Years** | **Location**  **Stage** | **Cohort** | **Comparison** | **Outcome** | **P Value** |
| --- | --- | --- | --- | --- | --- | --- | --- |
| Roxburgh^(36)^  2013 | 557  (150) | UK  2001-2010 | Colorectal  TNM I-IV | All cases | Tumour perforation | Association between > tumour perforation and Em | 0.010 |

*Supplementary Table 11 – Association between tumour perforation and mode of presentation*

| **Study** | **n – El**  **(n - Em)** | **Country**  **Years** | **Location**  **Stage** | **Cohort** | **Comparison** | **Outcome** | **P Value** |
| --- | --- | --- | --- | --- | --- | --- | --- |
| Ghazi^(13)^  2013 | 845  (129) | Sweden  2004-2006 | Colorectal  TNM I-IV | All resections | Perineural invasion | Association between > perineural invasion and Em | 0.001 |
| Amri^(43)^  2015 | 969  (102) | USA  2004-2011 | Colon  TNM I-IV | All resections | Perineural invasion | Association between > perineural invasion and Em | 0.005 |
| Wanis^(30)^  2018 | 1008  (157) | Canada  2006-2015 | Colon  TNM I-III | Curative resections | Perineural invasion | Association between > perineural invasion and Em | - |

*Supplementary Table 12 – Association between perineural invasion and mode of presentation*

| **Study** | **n – El**  **(n - Em)** | **Country**  **Years** | **Location**  **Stage** | **Cohort** | **Comparison** | **Outcome** | **P Value** |
| --- | --- | --- | --- | --- | --- | --- | --- |
| Ghazi^(13)^  2013 | 845  (129) | Sweden  2004-2006 | Colorectal  TNM I-IV | All resections | Tumour desmoplasia | Association between > desmoplasia and Em | P=0.03 |

*Supplementary Table 13 – Association between tumour desmoplasia and mode of presentation*

| **Study** | **n – El**  **(n - Em)** | **Country**  **Years** | **Location**  **Stage** | **Cohort** | **Comparison** | **Outcome** | **P Value** |
| --- | --- | --- | --- | --- | --- | --- | --- |
| Ghazi^(13)^  2013 | 845  (129) | Sweden  2004-2006 | Colorectal  TNM I-IV | All resections | Tumour necrosis | No significant different between El and Em | P=0.33 |

*Supplementary Table 14 – Association between tumour necrosis and mode of presentation*

| **Study** | **n – El**  **(n - Em)** | **Country**  **Years** | **Location**  **Stage** | **Cohort** | **Comparison** | **Outcome** | **P Value** |
| --- | --- | --- | --- | --- | --- | --- | --- |
| Ghazi^(13)^  2013 | 845  (129) | Sweden  2004-2006 | Colorectal  TNM I-IV | All resections | Tumour budding | No significant difference between El and Em | 0.28 |

*Supplementary Table 15* – Association between tumour budding and mode of presentation

| **Study** | **n – El**  **(n - Em)** | **Country**  **Years** | **Location**  **Stage** | **Cohort** | **Comparison** | **Outcome** | **P Value** |
| --- | --- | --- | --- | --- | --- | --- | --- |
| Ghazi^(13)^  2013 | 845  (129) | Sweden  2004-2006 | Colorectal  TNM I-IV | All resections | Mod-Well vs Poorly dif | No sig dif | 0.21 |
| Yang^(15)^  2011 | 1457  (215) | China  1998-2005 | Colorectal  TNM I-IV | All surgery | Well vs Mod vs Poorly dif | No sig dif | 0.396 |
| Gunnarsson^(25)^  2011 | 585  (97) | Sweden  1996-2005 | Colon  TNM I-IV | All resections | Low vs medium vs high grade | No sig dif | 0.4 |
| Mitchell^(33)^  2007 | 338  (105) | Canada  2002-2004 | Colorectal  TNM I-IV | All resections | Well vs Mod vs Poorly dif | No sig dif | 0.71 |
| Roxburgh^(36)^  2013 | 557  (113) | UK  2001-2010 | Colorectal  TNN I-IV | All cases | Well-mod vs poorly dif | Association between poorly dif and Em | 0.020 |
| Pruitt^(45)^  2014 | 50773  (20278) | USA  1992-1995 | Colorectal  TNM I-IV | All cases | Low vs high grade | Association between High Grade and Em | <0.001 |
| Biondo^(27)^  2005 | 194  (57) | Spain  1996-1998 | Colorectal  TNM I-III | Curative resections | Well vs Mod vs Undif | No sig dif | 0.660 |
| Bayar^(18)^  2016 | 230  (90) | Turkey  2009-2013 | Colorectal  TNM I-IV | All surgery | Well vs Mod vs Poorly dif | Association between >dif and Em | <0.001 |
| Okuda^(44)^  2018 | 449  (48) | Japan  2007-2011 | Colorectal  TNM II-III | Curative resections | Mod-well vs poorly dif TNM II | No sig dif | 1.000 |
|  |  |  |  |  | Mod-well vs poorly dif TNM III | No sig dif | 0.787 |
| Oliphant^(20)^  2014 | 1530  (228) | UK  2001-2004 | Colorectal  TNM I-II | Curative resections | Well-mod vs poorly dif | No sig dif | 0.103 |
| Hogan^(28)^  2015 | 342  (97) | Ireland  2000-2010 | Colon  TNM I-IV | Curative resections | Well dif em vs el | Assoc between <well dif and Em | 0.003 |
|  |  |  |  |  | Mod dif em vs el | No sig dif | 0.141 |
|  |  |  |  |  | Poorly dif em vs el | No sig dif | 0.164 |
| Wanis^(30)^  2018 | 1002  (156) | Canada  2006-2015 | Colon  TNM I-III | Curative resections | Poor/undif vs mod/well dif | No sig dif | - |
| Weixler^(7)^  2016 | 634  (77) | Switzerland  1989-2013 | Colorectal  TNM I-IV | All resections | G1 vs G2vs G3 | No sig dif | 0.180 |

*Supplementary Table 16 – Association between tumour grade/differentiation and mode of presentation*

| **Study** | **n – El**  **(n- Em)** | **Country**  **Years** | **Location**  **Stage** | **Cohort** | **Comparison** | **Outcome** | **P value** |
| --- | --- | --- | --- | --- | --- | --- | --- |
| Sucullu^(24)^  2015 | 186  (66) | Turkey  2004-2013 | Colon  TNM I-IV | Curative resections | M vs F | Association between >F and Em | 0.740 |
| Crozier^(46)^  2009 | 133  (55) | UK  1999-2006 | Colon  TNM I-III | Curative resections | M vs F | No sig dif | 0.268 |
| Scott^(47)^  1995 | 633  (272) | UK  1982-1992 | Colorectal  TNM I-III | All cases | M vs F | No sig dif | N.S. |
| Shah^(48)^  2013 | 457845  (54400) | USA  2003-2007 | Colorectal  TNM I-IV | All surgery | M vs F | Assoc between >M and Em | 0.017 |
| Yang^(15)^  2011 | 1457  (215) | China  1998-2005 | Colorectal  TNM I-IV | All surgery | M vs F | No sig dif | 0.407 |
| Askari^(16)^  2015 | 1648  (263) | UK  2004-2014 | Colorectal  TNM I-IV | All surgery | M vs F | No sig dif | 0.175 |
| Gunnarsson^(32)^  2013 | 9437  (2856) | Sweden  1997-2006 | Colon  TNM I-IV | All cases | M vs F | No sig dif | 0.125 |
| Gunnarsson^(25)^  2011 | 488  (97) | Sweden  1996-2005 | Colon  TNM I-IV | All resections | M vs F | No sig dif | 0.9 |
| Mitchell^(33)^  2007 | 347  (108) | Canada  2002-2004 | Colorectal  TNM I-IV | All resections | M vs F | Association between >F and Em | 0.004 |
| Pruitt^(45)^  2014 | 59082  (24248) | USA  1992-2005 | Colorectal  TNM I-IV | All cases | M vs F | Association between >F and Em | <0.001 |
| Borowski^(37)^  2016 | 916  (229) | UK  2009-2014 | Colorectal  TNM I-IV | All cases | M vs F | Association between >F and Em | 0.039 |
| Rabeneck^(49)^  2005 | 47564  (12106) | Canada  1993-2001 | Colorectal  TNM I-IV | All cases | M vs F | Association between >F and Em | <0.001 |
| Schneider^(50)^  2013 | 137  (52) | UK  2002-2004 | Colorectal  TNM I-IV | All cases | M vs F | No sig dif | 0.80 |
| Amri^(43)^  2015 | 969  (102) | USA  2004-2011 | Colon  TNM I-IV | All resections | M vs F | No sig dif | 0.51 |
| Biondo^(27)^  2005 | 207  (59) | Spain  1996-1998 | Colon  TNM I-III | Curative resections | M vs F | No sig dif | 0.760 |
| Bayar^(18)^  2016 | 230  (90) | Turkey  2009-2013 | Colorectal  TNM I-IV | All surgery | M vs F | No sig dif | 0.904 |
| Okuda^(44)^  2018 | 885  (94) | Japan  2007-2011 | Colorectal  TNM II-III | Curative resections | M vs F TNM II | No sig dif | 0.211 |
|  |  |  |  |  | M vs F TNM III | No sig dif | 0.679 |
| Oliphant^(20)^  2014 | 1626  (251) | UK  2001-2004 | Colorectal  TNM I-II | Curative resections | M vs F | No sig dif | 0.103 |
| Bakker^(29)^  2016 | 24960  (5947) | Netherlands  2009-2013 | Colon  TNM I-IV | All resections | M vs F | Association between >F and Em | - |
| Boeding^(22)^  2018 | 1058  (178) | Netherlands  2004-2015 | Colorectal  TNM I-IV | All resections | M vs F | No sig dif | 0.54 |
| Wanis^(30)^  2018 | 1022  (158) | Canada  2006-2015 | Colon  TNM I-III | Curative resections | M vs F | Association between >F and Em | - |
| Beuran^(41)^  2018 | 340  (275) | Romania  2011-2016 | Left colon  TNM I-IV | All resections | M vs F | No sig dif | >0.05 |
| Ho^(23)^  2010 | 1200  (225) | Australia  1984-2004 | Colorectal  TNM I-III | Curative resections | M vs F | No sig dif | 0.267 |
| Askari^(51)^  2017 | 216873  (69718) | UK  1997-2012 | Colorectal  TNM I-IV | All surgery | M vs F | Association between >F and Em | <0.001 |

*Supplementary Table 17* – Association between sex and mode of presentation

| **Study** | **n – El**  **(n- Em)** | **Country**  **Years** | **Location**  **Stage** | **Cohort** | **Comparison** | **Outcome** | **P Value** |
| --- | --- | --- | --- | --- | --- | --- | --- |
| Renzi^(52)^  2016 | 1158  (448) | UK  2005-2006 | Colorectal  TNM I-IV | All cases | Proportion 25-59/60-69/70-79/80+ | Association between > age and Em | 0.04 |
| Sucullu^(24)^  2015 | 186  (66) | Turkey  2004-2013 | Colon  TNM I-IV | Curative resections | Mean age | No sig dif | 0.3 |
| Crozier^(46)^  2009 | 133  (55) | UK  1999-2006 | Colon  TNM I-III | Curative resections | Proportion  <65/65-74/>75 | No sig dif | 0.204 |
| Rabeneck^(14)^  2005 | 33617  (7739) | Canada  1996-2001 | Colorectal  TNM I-IV | All cases | Proportion  20-49/50-69/>70 | Association between > age and Em | <0.001 |
| Scott^(47)^  1995 | 633  (272) | UK  1982-1992 | Colorectal  TNM I-III | All cases | Median age | Association between > age and Em | 0.04 |
| MacDonald^(53)^  2011 | 1223  (395) | UK  2006-2008 | Colorectal  TNM I-IV | All resections | Mean age | Association between > age and Em | <0.05 |
| Shah^(48)^  2013 | 453723  (54027) | USA  2003-2007 | Colorectal  TNM I-IV | All surgery | Proportion  <65/65+ | No sig dif | 0.455 |
| Yang^(15)^  2011 | 1672  (215) | China  1998-2005 | Colorectal  TNM I-IV | All surgery | Mean | No sig dif | 0.140 |
|  |  |  |  |  | <40/41-64/65+ | No sig dif | 0.487 |
| Anderson^(17)^  1992 | 363  (207) | UK  1974-1979 | Colorectal  TNM I-IV | All cases | Proportion  <55/55-64/65-74/75+ | Association between > age and Em | X |
| Gunnarsson^(32)^  2013 | 9437  (2856) | Sweden  1997-2006 | Colon  TNM I-IV | All cases | Proportion  <69/70-79/>80 | Association between > age and Em | <0.001 |
| Gunnarsson^(25)^  2011 | 488  (97) | Sweden  1996-2005 | Colon  TNM I-IV | All resections | Mean age | Association between > age and Em | 0.04 |
| Mitchell^(33)^  2007 | 347  (108) | Canada  2002-2004 | Colorectal  TNM I-IV | All resections | Mean age (years) | Association between > age and Em | 0.005 |
| Nascimbeni^(34)^  2008 | 430  (41) | Italy  1975-1984 | Colorectal  TNM I-IV | All surgery | Mean age | Association between > age and Em | 0.002 |
| Gunnarsson^(35)^  2014 | 508  (263) | Sweden  2006-2008 | Colon  TNM I-IV | All surgery | Proportion >85 +  Median age | No sig dif | 0.35 |
| Roxburgh^(36)^  2013 | 690  (187) | UK  2007-2010 | Colorectal  TNM I-IV | All cases | Proportion  <65/65-75/>75 | Association between > age and Em | <0.001 |
| Borowski^(37)^  2016 | 916  (229) | UK  2009-2014 | Colorectal  TNM I-IV | All cases | Median age | No sig dif | - |
| Catena^(39)^  2009 | 56  (50) | Italy  1991-2001 | Colon/high rectal  TNM I-IV | All resections | Mean age | No sig dif | >0.05 |
| Amri^(43)^  2015 | 969  (102) | USA  2004-2011 | Colon  TNM I-IV | All resections | Median age | No sig dif | 0.24 |
| Biondo^(27)^  2005 | 207  (59) | Spain  1996-1998 | Colon  TNM I-III | Curative resections | Mean age | No sig dif | 0.900 |
| Bayar^(18)^  2016 | 230  (90) | Turkey  2009-2013 | Colorectal  TNM I-IV | All surgery | Mean age | No sig dif | >0.05 |
| Sikka^(5)^  2012 | 6938  (2092) | USA  1996-2000 | Colorectal  TNM I-IV | All cases | Proportion 66-69/70-74/75-79/80-84/>85 | Association between > age and Em | X |
| Okuda^(44)^  2018 | 885  (94) | Japan  2007-2011 | Colorectal  TNM II-III | Curative resections | Median age TNM II | No sig dif | 0.683 |
|  |  |  |  |  | Median age TNM III | No sig dif | 0.058 |
| McArdle^(19)^  2004 | 2214  (986) | UK  1991-1994 | Colorectal  TNM I-IV | All resections | Proportion  <64/65-74/>75 | Association between > age and Em | <0.001 |
| Oliphant^(20)^  2014 | 1626  (251) | UK  2001-2004 | Colorectal  TNM I-III | Curative resections | Mean age | Association between > age and Em | 0.023 |
| Kundes^(40)^  2016 | 209  (51) | Turkey  2012-2014 | Colorectal  TNM I-II | Curative resections | Mean age | Association between > age and Em | 0.02 |
| Bakker^(29)^  2016 | 24960  (5947) | Netherlands  2009-2013 | Colon  TNM I-IV | All resections | Proportion  <75/75+ | Association between > age and Em | X |
| Wanis^(30)^  2018 | 1022  (158) | Canada  2006-2015 | Colon  TNM I-III | All resections | Mean age | Association between > age and Em | X |
| Beuran^(41)^  2018 | 340  (275) | Romania  2011-2016 | Left colon  TNM I-IV | All resections | Mean age | No sig dif | 0.102 |
| Askari^(51)^  2017 | 216873  (69718) | UK  1997-2012 | Colorectal  TNM I-IV | All surgery | Proportion  18-54/55-69/70/79/>79 | Association between age <55 and >79 and Em | <0.001 |

*Supplementary Table 18* – Association between age and mode of presentation

| **Study** | **n – El**  **(n - Em)** | **Country**  **Years** | **Location**  **Stage** | **Cohort** | **Comparison** | **Outcome** | **P Value** |
| --- | --- | --- | --- | --- | --- | --- | --- |
| Pruitt^(45)^  2014 | 59082  (24248) | USA  1992-2005 | Colorectal  TNM I-IV | All cases | African Americans vs Whites | Association between > non-white and Em | <0.05 |
| Amri^(43)^  2015 | 969  (102) | USA  2004-2011 | Colon  TNM I-IV | All resections | Ethnic minority yes vs no | No sig dif | 0.23 |
| Sikka^(5)^  2012 | 6938  (2092) | USA  1996-2000 | Colorectal  TNM I-IV | All cases | White vs African American | Association between > non-white and Em | <0.05 |
| Askari^(51)^  2017 | 41048  (15512) | UK  1997-2012 | Colorectal TNM I-IV | All surgery | White vs Black vs Asian | Association between > non-white and Em | <0.001 |

*Supplementary Table 19 – Association between ethnicity and mode of presentation*

| **Study** | **n – El**  **(n - Em)** | **Country**  **Years** | **Location**  **Stage** | **Cohort** | **Comparison** | **Outcome** | **P Value** |
| --- | --- | --- | --- | --- | --- | --- | --- |
| Costa^(54)^  2017 | 87  (87) | Italy  2006-2012 | Colorectal  TNM I-IV | All resections | Median BMI | No sig dif | 0.09 |
| Mitchell^(33)^  2007 | 347  (108) | Canada  2002-2004 | Colorectal  TNM I-IV | All resections | BMI (categories) | Association between BMI >40/<25 and Em | 0.001 |
| Amri^(43)^  2015 | 969  (102) | USA  2004-2011 | Colon  TNM I-IV | All resections | Median BMI | No sig dif | 0.29 |

*Supplementary Table 20 – Association between body mass index and mode of presentation*

| **Study** | **n – El**  **(n - Em)** | **Country**  **Years** | **Location**  **Stage** | **Cohort** | **Comparison** | **Outcome** | **P Value** |
| --- | --- | --- | --- | --- | --- | --- | --- |
| Blind^(55)^  2018 | 304  (76) | Sweden  2007-2010 | Colon  TNM I-IV | All surgery | Mean distance | No sig dif | 0.433 |
|  |  |  |  |  | Distance quartiles | No sig dif | >0.05 |

*Supplementary Table 21 – Association between distance to hospital and mode of presentation*

| **Study** | **n – El**  **(n - Em)** | **Country**  **Years** | **Location**  **Stage** | **Cohort** | **Comparison** | **Outcome** | **P Value** |
| --- | --- | --- | --- | --- | --- | --- | --- |
| Crozier^(46)^  2009 | 133  (55) | UK  1996-2006 | Colon  TNM I-III | Curative resections | Carstairs  1-2/3-5/6-7 | No sig dif | 0.142 |
| Rabeneck^(14)^  2006 | 32779 (7378) | Canada  1996-2001 | Colorectal  TNM I-IV | All cases | Income quintile | Assoc between > deprivation and Em | <0.001 |
| Scott^(47)^  1995 | 333  (117) | UK  1982-1992 | Colorectal  TNM I-III | All cases | Social class  1-2/4-7 | No sig dif | >0.05 |
| Askari^(16)^  2015 | 1607  (257) | UK  2004-2014 | Colorectal  TNM I-IV | All surgery | IMD quintile | No sig dif | 0.444 |
| Oliphant^(56)^  2013 | 3351  (945) | UK  2001-2004 | Colorectal  TNM I-IV | All surgery | SIMD quintile | Assoc between > deprivation and Em | 0.033 |
| Blind^(55)^  2018 | 304  (76) | Sweden  2007-2010 | Colon  TNM I-IV | All surgery | Average income  High/low | No sig dif | 0.122 |
| A El^(57)^  2016 | 434  (99) | UK  2010-2014 | Colorectal  TNM I-IV | All cases | IMD quintile | No sig dif | 0.10 |
| Gunnarsson^(32)^  2013 | 9420 (2850) | Sweden  1997-2006 | Colon  TNM I-IV | All cases | Education | Assoc between > deprivation and Em | 0.018 |
|  |  |  |  |  | Income quartile | Assoc between > deprivation and Em | <0.001 |
| Mitchell^(33)^  2007 | 347  (108) | Canada  2002-2004 | Colorectal  TNM I-IV | All resections | Income quintile | No sig dif | 0.82 |
|  |  |  |  |  | Education level | No sig dif | 0.46 |
| Hole^(58)^  2002 | 1545  (724) | UK  1991-1994 | Colorectal  TNM I-IV | All resections | Carstairs  1-2/3-5/6-7 | No sig dif | 0.80 |
| Roxburgh^(36)^  2013 | 690  (187) | UK  2001-2010 | Colorectal  TNM I-IV | All cases | Carstairs  1-2/3-4/5-6 | No sig dif | 0.384 |
| Pruitt^(45)^  2014 | 59082 (24248) | USA  1992-2005 | Colorectal  TNM I-IV | All cases | Neighbourhood poverty rate | Assoc between > deprivation and Em | <0.0001 |
| Borowski^(37)^  2016 | 1145  (915) | UK  2009-2014 | Colorectal  TNM I-IV | All cases | LSOA quintiles | Assoc between > deprivation and Em | 0.048 |
| Askari^(51)^  2017 | 215097 (69138) | UK  1997-2012 | Colorectal  TNM I-IV | All surgery | IMD quintile | Assoc between > deprivation and Em | <0.001 |

*Supplementary Table 22– Association between socioeconomic status and mode of presentation*

| **Study** | **n – El**  **(n - Em)** | **Country**  **Years** | **Location**  **Stage** | **Cohort** | **Comparison** | **Outcome** | **P Value** |
| --- | --- | --- | --- | --- | --- | --- | --- |
| Catena^(39)^  2009 | 56  (50) | Italy  1999-2001 | Colon and high rectal  TNM I-IV | All resections | ASA grade (1/2/3/4) | Assoc between > ASA and Em | P<0.05 |
| Bakker^(29)^  2016 | 24960 (5947) | Netherlands  2009-2013 | Colon  TNM I-IV | All resections | ASA grade 1-2 vs 3+ | Assoc between >ASA and Em | - |
| Ming-Gao^(42)^  2014 | 261  (85) | China  2000-2010 | Colorectal  TNM I-IV | All surgery | ASA grade | Assoc between >ASA and Em | P<0.01 |

*Supplementary Table 23 - Association between ASA grade and mode of presentation*

| **Study** | **n – El**  **(n - Em)** | **Country**  **Years** | **Location**  **Stage** | **Cohort** | **Comparison** | **Outcome** | **P Value** |
| --- | --- | --- | --- | --- | --- | --- | --- |
| Rabeneck^(14)^  2006 | 33617 (7739) | Canada  1996-2001 | Colorectal  TNM I-IV | All cases | Deyo score | Assoc between > comorbidity and Em | <0.001 |
| Shah^(48)^  2013 | 454000 (54032) | USA  2003-2007 | Colorectal  TNM I-IV | All surgery | Charlson score | Assoc between < comorbidity and Em | P=0.000 |
| Yang^(15)^  2011 | 1457  (215) | China  1998-2005 | Colorectal  TNM I-IV | All surgery | Number of comorbidities | Assoc between > comorbidity and Em | P=0.002 |
| Askari^(16)^  2015 | 1647  (264) | UK  2004-2014 | Colorectal  TNM I-IV | All surgery | Charlson score Em vs El | Assoc between > comorbidity and Em | <0.001 |
| Gunnarsson ^(35)^  2014 | 577  (263) | Sweden  2006-2008 | Colon  TNM I-IV | All surgery | CV disease Diabetes Resp disease | No sig dif | P=0.34/0.12/0.39 |
| Wallace^(59)^  2014 | 64884 (17889) | UK  2007-2011 | Colorectal  TNM I-IV | All cases | Individual components of Charlson Score | Assoc between > comorbidity and Em | Not provided |
| Amri^(43)^  2015 | 969  (102) | USA  2004-2011 | Colon  TNM I-IV | All resections | Charlson score | No sig dif | 0.62 |
|  |  |  |  |  | DM Type 2 | No sig dif | 0.96 |
|  |  |  |  |  | IBD | No sig dif | 0.36 |
|  |  |  |  |  | Prev colorectal cancer | No sig dif | 0.30 |
|  |  |  |  |  | Prev polyps | No sig dif | 0.56 |
| Bayar^(18)^  2016 | 230  (90) | Turkey  2009-2013 | Colorectal  TNM I-IV | All surgery | Yes/no | Assoc between >comorbidity and Em | P<0.001 |
| Sikka^(5)^  2012 | 6938  (2092) | USA  1996-2010 | Colorectal  TNM I-IV | All cases | Charlson score | Assoc between > comorbidity and Em | P<0.05 |
| Bakker^(29)^  2016 | 24960 (5947) | Netherlands  2009-2013 | Colon  TNM I-IV | All resections | Charlson score 0/1/2+ | Assoc between < comorbidity and Em | Not provided |
| Neuman^(60)^  2013 | 31574 (14650) | USA  1992-2005 | Colon  TNM I-IV | All cases | Individual co-morbidities | Assoc between > comorbidity and Em | P<0.001 |

*Supplementary Table 24 – Association between other assessments of co-morbidity and mode of presentation*

| **Study** | **n – El**  **(n - Em)** | **Country**  **Years** | **Location**  **Stage** | **Cohort** | **Comparison** | **Outcome** | **P Value** |
| --- | --- | --- | --- | --- | --- | --- | --- |
| Park^(61)^  2018 | 1077  (63) | UK/Japan  1997-2013  2005-2015 | Colorectal  TNM I-III | Curative resections | mGPS | Association between > mGPS and Em | <0.001 |
|  |  |  |  |  | NLR | Association between > NLR and Em | <0.001 |
| Catena^(39)^  2009 | 56  50 | Italy  1999-2001 | Colon and high rectal  TNM I-IV | All resections | Median CRP | Association between > CRP and Em | <0.05 |

*Supplementary Table 25 – Association between preoperative systemic inflammatory response and mode of presentation*

| **Study** | **n – El**  **(n - Em)** | **Country**  **Years** | **Location**  **Stage** | **Cohort** | **Comparison** | **Outcome** | **P Value** |
| --- | --- | --- | --- | --- | --- | --- | --- |
| Gunnarsson^(25)^  2011 | 482  (97) | Sweden  1996-2005 | Colon  TNM I-IV | All resections | Seasonal change Em vs El | More Em cases in summer  No sig association between El and seasons | 0.05 |

*Supplementary Table 26 – Association between time of year and mode of presentation*

| **Study** | **n – El**  **(n - Em)** | **Country**  **Years** | **Location**  **Stage** | **Cohort** | **Comparison** | **Outcome** | **P Value** |
| --- | --- | --- | --- | --- | --- | --- | --- |
| Cleary^(64)^  2007 | 310 (62) | UK  1998-2002 | Colorectal  TNM I-IV | All cases | Haemoglobin  <13 | Association between < Hb and Em | <0.001 |
|  |  |  |  |  | Weight loss | Association between > weight loss and Em | <0.001 |

*Supplementary Table 27 – Association between Haemoglobin and weight loss and mode of presentation*

| **Study** | **n – El**  **(n -Em)** | **Country**  **Years** | **Location**  **Stage** | **Cohort** | **Comparison** | **Outcome** | **P Value** |
| --- | --- | --- | --- | --- | --- | --- | --- |
| Catena^(39)^  2009 | 56  (50) | Italy  1999-2001 | Colon and high rectal  TNM I-IV | All resections | Preop CEA | Assoc between >CEA and Em | <0.05 |
|  |  |  |  |  | Preop TNF A | No sig dif | Non sig |
|  |  |  |  |  | Preop and postop IL1 | Assoc between >IL1 and Em | <0.05 |
|  |  |  |  |  | Preop and postop IL6 | Assoc between >IL6 and Em | <0.05 |

*Supplementary Table 28 – Association between: CEA, TNF A, IL1 and IL6 and mode of presentation*
